# Multimodal MR imaging signatures to identify brain diffuse midline gliomas with H3 K27M mutation

**DOI:** 10.1002/cam4.4500

**Published:** 2021-12-24

**Authors:** Xiaorui Su, Yanhui Liu, Haoyu Wang, Ni Chen, Huaiqiang Sun, Xibiao Yang, Weina Wang, Simin Zhang, Xinyue Wan, Qiaoyue Tan, Qiang Yue, Qiyong Gong

**Affiliations:** ^1^ Huaxi MR Research Center (HMRRC) Department of Radiology West China Hospital of Sichuan University Chengdu China; ^2^ Huaxi Glioma Center West China Hospital of Sichuan University Chengdu China; ^3^ Research Unit of Psychoradiology Chinese Academy of Medical Sciences Chengdu China; ^4^ Department of Neurosurgery West China Hospital of Sichuan University Chengdu China; ^5^ Department of Pathology West China Hospital of Sichuan University Chengdu China; ^6^ Functional and Molecular Imaging Key Laboratory of Sichuan Province Chengdu China; ^7^ Department of Radiology West China Hospital of Sichuan University Chengdu China; ^8^ Department of Radiology The First Affiliated Hospital College of Medicine Zhejiang University Hangzhou Zhejiang China; ^9^ Department of Radiotherapy West China Hospital of Sichuan University Chengdu China

**Keywords:** functional magnetic resonance imaging, glioma, histone H3.3, quantitative evaluation

## Abstract

**Background:**

Conventional MR imaging has limited value in identifying H3 K27M mutations. We aimed to investigate the capacity of quantitative MR imaging variables in identifying the H3 K27M mutation status of diffuse midline glioma.

**Materials and Methods:**

Twenty‐three patients with H3 K27M mutation and thirty‐two wild‐type patients were recruited in this retrospective study, all of whom underwent multimodal MR imaging. Clinical data and quantitative MR imaging variables were explored by subgroup analysis stratified by age (juveniles and adults). Then, a logistic model for all patients was constructed to identify potential variables for predicting K27M mutation status. Besides, a retrospective validation set including 13 patients was recruited. The C‐index and F1 score were used to evaluate the performance of the prediction model.

**Results:**

It turned out that patients with H3 K27M mutation were younger in the adult subgroup. In the mutation group, some relative apparent diffusion coefficient (rADC) histogram parameters and myo‐inositol/creatine plus phosphocreatine (Ins/tCr) ratio were lower than in the wild‐type group of both juveniles and adults (*p* < 0.05). After nested cross‐validation and LASSO algorithm, the age, Ins/tCr, and rADC_15th were selected as potential predictors for H3 K27M mutation in the model. The nomogram model showed good diagnostic power with a validated C‐index of 0.884. In addition, the area under the curve (AUC) was 0.898 (0.976 in validation set) and the F1 score was 0.732.

**Conclusions:**

In conclusion, age, rADC_15th, and Ins/tCr values were helpful in identifying H3 K27M mutations in midline gliomas.

## Graphical abstract

(1) Quantitative images features were applied to identify H3 K27M mutation status. (2) Age, rADC_15th, and Ins/tCr values were most helpful in identifying H3 K27M status. (3) The model showed good diagnostic power with AUC of 0.898 and F1 score of 0.732.

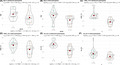

## 1 INTRODUCTION

An H3 K27M mutation is detected as a lysine to methionine substitution at codon 27 in the H3F3A gene or in the HIST1H3B/C gene, and is designated as a molecular marker to define a tumor entity in the 2016 World Health Organization Classification of Tumours of the Central Nervous System.^1^ These mutations are often present in gliomas located in midline structures of the brain, such as the brain stem, thalamus, and hypothalamus.^2^ Gliomas in the brainstem are also named as diffuse intrinsic pontine gliomas (DIPGs), nearly 78% of which carry H3 K27M mutations.^3^ Midline gliomas with H3 K27M mutations were found to be a subgroup with poor clinical prognosis independent of tumor location.^4^, ^5^

The clinical and imaging characteristics of gliomas with H3 K27M mutations have been described in some previous studies, not only in pediatric patients, but also in adult populations,^6^, ^7^, ^8^, ^9^ with a frequency of H3 K27M mutations of approximately 13% in adult patients.^9^ According to these studies, gliomas with an H3 K27M mutation showed large histological discrepancies as well as variable radiographic features.^6^, ^7^, ^8^ In a study of pediatric patients with diffuse midline gliomas, MR imaging showed that these gliomas had a diverse appearance and did not show distinguishing features between the mutation group and the wild‐type group.^2^ However, most radiological studies were based on conventional imaging and only described tumor location, pattern of contrast enhancement, peritumoral edema, etc., and thus were less informative for proper evaluation of tumor characteristics. A pilot study including 14 midline glioma patients with K27M mutations reported that quantitative metrics, that is, the maximal tumor‐to‐background ratio (TBRmax) in ^18^F‐FET‐PET, could be used to visualize tumor progression.^10^ This may also shed a light on the application of quantitative MR imaging parameters to differentiate gliomas with an H3 K27M mutation from their wild‐type counterparts.

Advanced functional MR imaging techniques have proved to be powerful tools to noninvasively characterize brain lesions in vivo.^11^ We hypothesized that the quantitative MR characteristics might be useful for identifying the H3 K27M mutation status of midline gliomas, and may compensate for the weakness of conventional MRI (magnetic resonance imaging). In the present study, we applied multimodal, functional MR imaging including diffusion‐weighted imaging (DWI), diffusion‐tensor imaging (DTI), perfusion‐weighted imaging (PWI, dynamic susceptibility contrast imaging was used in our study) and magnetic resonance spectroscopy (MRS) to explore the quantitative imaging characteristics of midline gliomas with and without an H3 K27M mutation. Specifically, we applied subgroup analysis to explore clinical and imaging features in juvenile and adult patients, separately. Additionally, a logistic model was established to select the most valuable imaging or clinical variables for identifying H3 K27M status.

## 2 MATERIALS AND METHODS

### 2.1 Participants

**FIGURE 1:**
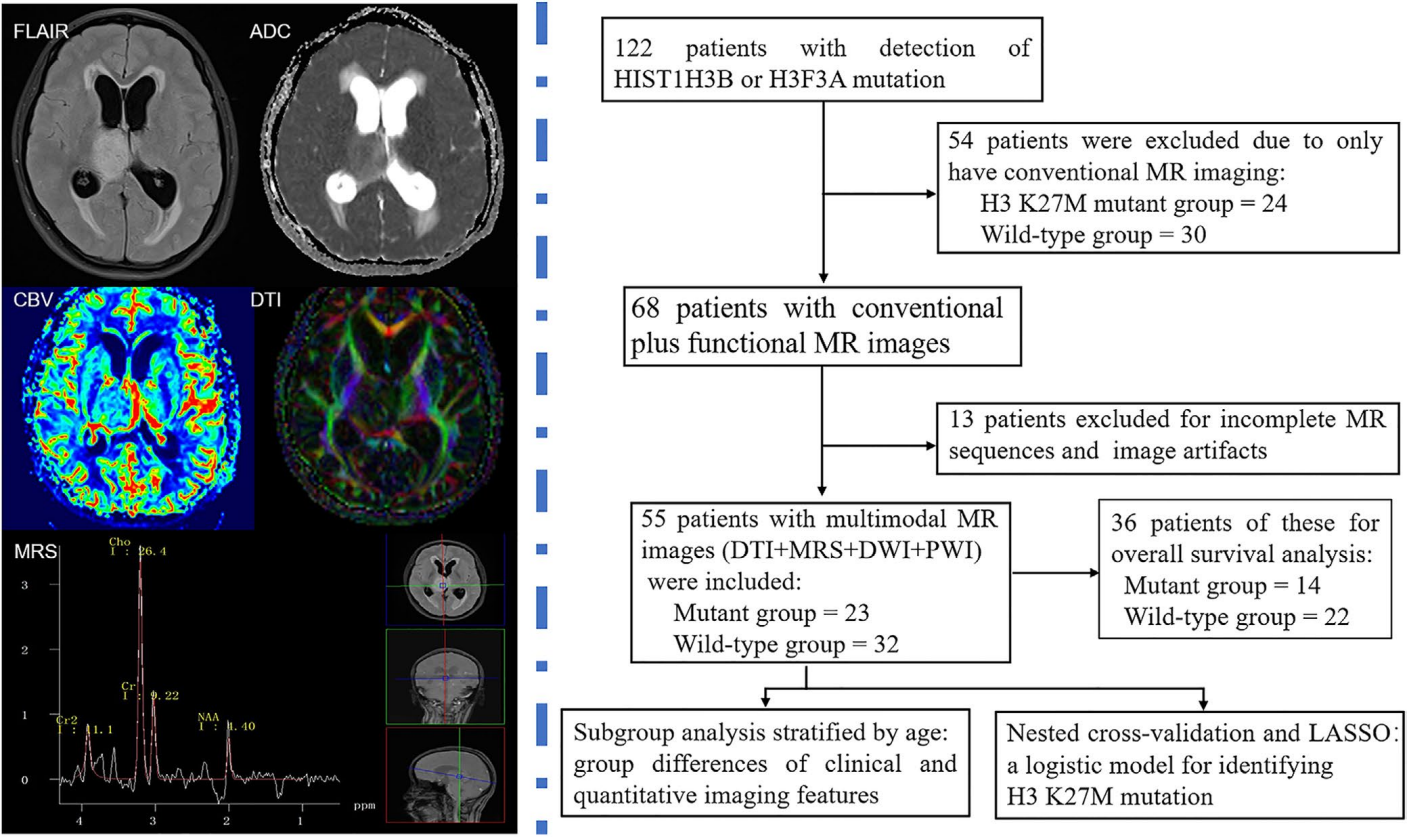
The workflow of this study

This study was approved by the Institutional Review Board (IRB) of West China Hospital of Sichuan University, and the requirement for written informed consent was waived. One hundred and twenty‐two patients with midline glioma with an H3F3A or a HIST1H3B mutation by pyrosequencing analysis^12^ were recruited retrospectively from our hospital between December 2016 and May 2020. All patients underwent conventional MR imaging and functional MR imaging, including DWI, DTI, MRS, and PWI. Sixty‐seven patients were excluded for incomplete functional MRI data or poor image quality. Finally, 32 patients of the wild‐type group including 16 males (age range, 9–76 years) and 16 females (age range, 6–63 years) and 23 patients of the mutation group including 13 males (age range, 6–48 years) and 10 females (age range, 4–47 years) were enrolled in this study (see the flowchart in Figure 1). Patients’ illness duration and Karnofsky performance score (KPS) at diagnosis were also obtained. All patients were followed up after the first surgical resection for further overall survival (OS) analysis.

Moreover, a retrospective validation was performed on an independent patient set. We recruited new patients as a validation set from West China Hospital of Sichuan University between June 2020 and October 2021. Finally, six patients carrying the H3 K27M mutation (4–33 years, male/female = 3/3) and seven patients (23–62 years, male/female = 5/2) carrying the wild‐type sequence were included in the validation set.

### 2.2 MR image acquisition

All patients underwent MR examinations of the brain on a 3.0‐T clinical scanner with a 20‐channel head‐neck phased array head coil (Skyra, Siemens Healthcare). The major MR imaging protocols and parameters were as follows: (a) fast T2‐weighted spin‐echo with fat‐suppression (T2W_FS, repetition time/echo time [TR/TE] = 4500/105 ms) and axial T2‐weighted fluid‐attenuated inversion recovery (FLAIR, TR/TE = 6000/81 ms) images acquired for tumor identification, slice thickness = 5 mm; (b) 3D T1‐weighted magnetization‐prepared rapid acquisition gradient echo (T1_MPRAGE) parameters were sagittal sections = 176, TR/TE = 1630/2.3 ms, flip angle = 8°, slice thickness = 1 mm, and matrix = 256 × 232; (c) DWI parameters were axial sections = 42, TR/TE = 5300/102 ms, slice thickness = 5 mm, matrix = 192 × 192, 30 weighted diffusion acquisitions (*b* = 1000 sec/mm^2^), and one unweighted acquisition (*b* = 0 sec/mm^2^); (d) DTI parameters were axial sections = 62, TR/TE = 6000/93 ms, slice thickness = 3 mm, matrix = 128 × 128, *b* = 0, and 1000 sec/mm^2^; (e) PWI was acquired after a bolus injection of gadolinium‐based contrast agent (5 ml/s, 0.1 mmol/kg body weight), phase images = 60, axial sections = 1260, TR/TE = 1640/30 ms, flip angle = 90°, slice thickness = 5 mm, matrix = 128 × 128, and FOV = 22 cm × 22 cm, followed by post‐contrast T1_MPRAGE; (f) MRS (CSI_slaser_135) parameters were slice thickness = 3 mm, TR/TE = 1700/135, NEX = 1, FOV = 16 cm × 16 cm, volume of interest = 8 cm × 8 cm, and each voxel size = 1.0 × 1.0 × 1.0 = 1.0 ml.

### 2.3 Comparison of multimodal characteristics

First, we determined the tumor size by measuring the two longest perpendicular diameters of the hyperintense area on FLAIR images.^13^ Then, for multimodal MR image analysis, tumors were drawn as regions of interest (ROIs) based on FLAIR images to evaluate the quantitative MR metrics of the whole tumor. All ROIs were drawn on ITK‐SNAP^14^ (version 3.6.0, www.itksnap.org) by S.M.Z and X.R.S. with 3 and 5 years of clinical experience in diagnostic neuroradiology, respectively. A single normal ROI was drawn in the healthy contralateral brain or semiovale center when the tumor was occupied the entire brain stem, with areas ranging from 100 to 150 mm^2^. Both authors were blinded to the H3 K27M mutation status. The final ROIs were the overlapping volumes generated by two authors, and the ROIs with an overlapping rate <80% were reviewed and modified by a neuroradiologist with 21 years of clinical experience (Q.Y.).^15^ The intraclass correlation coefficient (ICC) for ROIs was 0.97 [95% CI, 0.96, 0.98]. The tumor ROIs were registered to PWI, DWI, and DTI images using FMRIB Software Library tools (FSL tools, version 6.03, www.fmrib.ox.ac.uk/fsl),^16^ and corresponding MR parameters were obtained for tumor ROIs as well as normal ROIs. Then, relative MR parameters were calculated as ratios of tumor/normal ROIs. For perfusion images, the values of relative cerebral blood volume (rCBV), relative cerebral blood flow (rCBF), relative mean transmit time (rMTT), and relative time to peak (rTTP) ratios were calculated. In addition, the relative fractional anisotropy (rFA) and relative mean diffusivity (rMD) based on DTI images and the percent based on ADC maps were also calculated. The FA and MD values were calculated by the *FDT diffusion* function after eddy current correction was performed using the FSL. In addition, histogram analysis (for nonzero voxels) was analyzed using *fslstats* in FSL, and the main histogram features included the mean, 25th percentile, 50th percentile, 75th percentile, and 100th percentile for all ADC, DTI, and PWI parameters; an additional 15th percentile for rADC^17^; and the 90th percentile for rCBV.^18^ For MRS, raw spectral data with a signal‐to‐noise ratio (SNR) for Cr >20 and a full width at half maximum <0.08 ppm were processed with LCModel (version 6.3‐1H) to obtain N‐acetylaspartate (NAA)/creatine plus phosphocreatine (tCr), NAA plus N‐acetylaspartylglutamate (tNAA)/tCr, glutamine plus glutamate (Glx)/tCr, choline (Cho)/tCr, Cho/NAA, and myo‐inositol (Ins)/tCr values. Standard error estimates (%SD) >20% were discarded.

### 2.4 Predictive analysis

Due to the relatively small sample size, at first, we applied nested cross‐validation (five‐folds) with random forest (“*caret*” package in R, R Version 3.6, http://www.r‐project.org) to avoid overfitting problems and reduce dimensions of data of all patients. In this step, we constructed five separate models to select several most important variables, according to the importance score (0–100) given by models. Second, to further select the most valuable features and to establish the prediction model for the H3 K27M mutation, the absolute shrinkage and selection operator (LASSO) binomial regression model available in the “*glmnet*” package was used. Finally, a nomogram was built by variables selected by the LASSO model using the “*rms*” package, and receiver operator characteristic curve (ROC) and precision‐recall curve were drawn.

### 2.5 Statistical analysis

The intergroup comparisons of clinical demographics and imaging features were based on subgroup analysis, including juvenile subgroup (age < 18 years old) and adult subgroup (age ≥ 18 years old), and each subgroup contained patients with H3 K27M mutant or wild‐type gliomas. The normality for each MR value and clinical characteristics, which were continuous variables, were examined by the Shapiro–Wilk test. The chi‐squared test was used to calculate intergroup differences in sex. A two‐sample *t* test or a nonparametric Wilcoxon rank sum test was used to examine the group effect using “*ggstatsplot*”, and Kaplan–Meier survival analysis was performed using “*survival*” in R software.

During nested cross‐validation process, feature importance of each model was generated by “*caret*” package. As for the LASSO model, the C‐index served as the performance estimator using “*Hmisc*” in R software and was validated using bootstrap resampling.^19^ Bootstraps with 200 resamples were used to calculate a corrected C‐index.^20^ F1 score, which was based on a balance between precision and recall curve,^21^ and the area under the curve (AUC) of the ROC were calculated to evaluate model performance regarding a differential diagnosis between the two groups, using “*pROC*”, “*InformationValue*”, and “*precrec*” packages, respectively in R software. During data processing, the “*tidyverse”* and “*data.table*” package were often used. A *p* < 0.05 was considered to be significant.

## 3 RESULTS

### 3.1 Patient demographics

**FIGURE 2:**
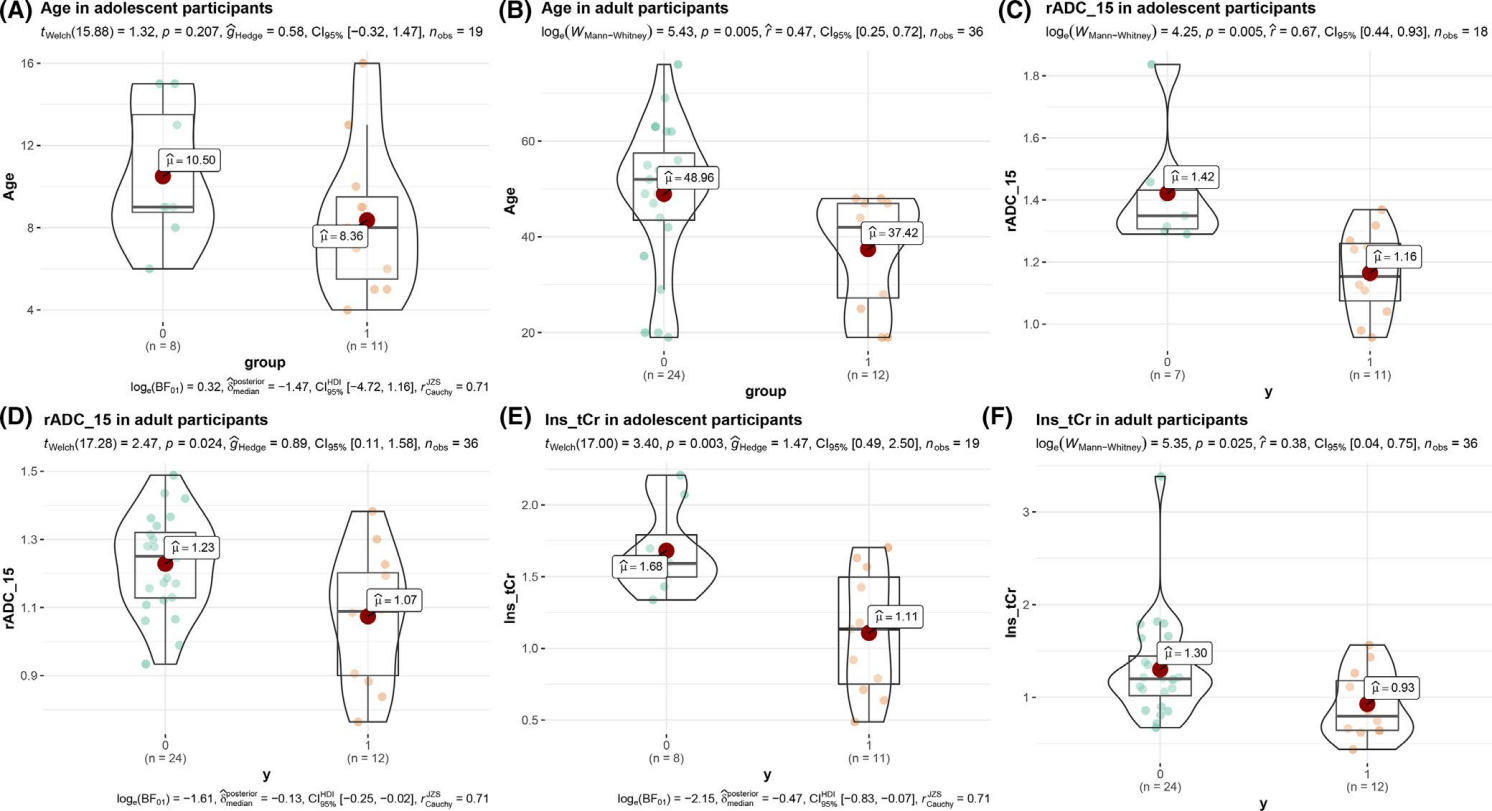
The quantitative variables with significant differences between the H3 K27M mutation group and the wild‐type group, including the age of the patients, the 15th percentile relative apparent diffusion coefficient (rADC) of diffusion‐weighted imaging (DWI), and myo‐inositol (Ins) to creatine plus phosphocreatine (tCr) ratio of MRS

There were 55 patients with midline glioma of the brain, including 32 wild‐type patients without H3 K27M mutation and 23 patients with H3 K27M wild‐type. As shown in Table 1, the whole cohort was separated into two subgroups: the juvenile subgroup and the adult subgroup. Patients with H3 K27M mutant gliomas were younger than those with wild‐type gliomas in the adult subgroup (mean age 37.41 years vs. 48.96 years, *p* = 0.005) (see Figure 2B), though not in the juvenile subgroup (mean age 8.36 years vs. 10.5 years, *p* = 0.21) (see Figure 2A). For both subgroups, there were no significant difference in sex, KPS, or illness duration between the mutation and the wild‐type group (*p* > 0.05). In the mutation group, most tumors were located in the brainstem, not only in juvenile patients (8/11, 72.7%), but also in adult patients (9/12, 75%, *p* < 0.01). In the wild‐type group, tumors tended to locate in the brainstem of juvenile patients (37.5%), while they tended to locate in diencephalon or hemisphere near the midline of adult patients (both 45.8%).

**TABLE 1:** The clinical characteristics of patients with diffuse midline gliomas

Variables	H3 K27M wild‐type glioma group	H3 K27M mutant glioma group	Statistic	*p* value
All participants	32	23		NA
Participants’ age <18 years	8	11		NA
KPS	82.5 ± 7.07	79.09 ± 8.31	*W* = 54.5	0.38
Illness_duration	1.4 ± 1.4	2.98 ± 3.53	*W* = 26.5	0.16
Male/female	4/4	4/7	NA	0.65^a^
Age (years)	10.5 ± 3.38	8.36 ± 3.64	1.32	0.21
Location, *n* (%)			NA	0.41
Hemisphere near midline	2 (25%)	1 (9.1%)		NA
Diencephalon	2 (25%)	2 (18.2%)		NA
Brainstem	3 (37.5%)	8 (72.7%)		NA
Cerebellum	1 (12.5%)	0 (0)		NA
Participants’ age ≥18 years	24	12		NA
KPS	78.26 ± 7.77	76.67 ± 6.51	*W* = 152	0.61
Illness_duration (month)	3.27 ± 3.4	2.9 ± 3.0	*W* = 135	0.93
Male/female	12/12	3/9	1.1571^#^	0.28
Age (years)	48.96 ± 15.1	37.41 ± 11.36	*W* = 227.5	**0.005** *
Location, *n* (%)			NA	<0.01*
Hemisphere near midline	11 (45.8%)	0 (0)		NA
Diencephalon	11 (45.8%)	3 (25%)		NA
Brainstem	1 (4.2%)	9 (75%)		NA
Cerebellum	1 (4.2%)	0 (0)		NA

The bold values means *p* < 0.05.

Abbreviations: KPS, Karnofsky performance status; NA, not applicable.

* *p* < 0.05; ^#^chi‐squared; ^a^Fisher test; *W*, Wilcoxon test statistic.

**FIGURE 3:**
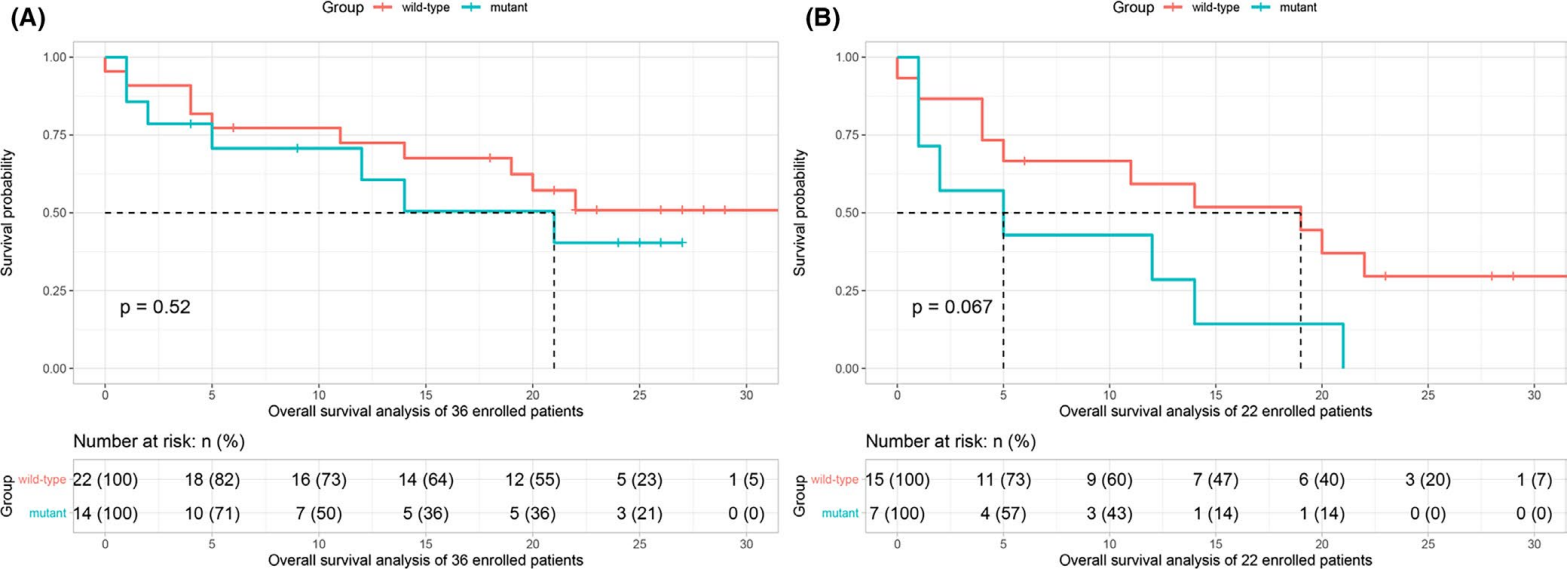
(A) Overall survival analysis of 36 patients including 18 wild‐type gliomas and 14 H3 K27M mutation gliomas, of which 14 patients were lost to follow‐up in their second or further follow‐up session; (B) Overall survival analysis of 22 patients with exact dead or alive status, including 15 wild‐type patients and seven H3 K27M mutation patients

For survival analysis, follow‐up data were obtained from 36 patients, but only 22 patients had exact survival status (dead or alive). Fourteen patients were lost to follow‐up in their second or further follow‐up session. For the 36 patients, there were no significant difference of median OS between the two groups (wild‐type group vs. mutation group, NA vs. 21 months, *p* = 0.52, see Figure 3A). However, survival analysis for the 22 patients showed that the mutation group displayed a trend of relatively poor outcomes with a median OS of 5 months compared with the wild‐type group which had a median OS of 19 months (*p* = 0.067, see Figure 3B).

### 3.2 Comparison of multimodal radiographical characteristics

During the subgroup comparison of multimodal MR imaging variables, differences in several quantitative MR metrics were noted between the H3 K27M mutation group and the wild‐type group (DWI and MRS results were shown in Table 2, others in supplemental Table S1). Both subgroup analyses indicated that rADC_15th, rADC_25th, rADC_50th, and rADC_75th values of the mutation group were significantly lower than those of the wild‐type group (*p* < 0.05). In addition, lower Ins/tCr values were detected in the juvenile (*p* = 0.003) and the adult (*p* = 0.025) mutation subgroups compared with the wild‐type subgroups (Figure 2E,F). However, no significant difference in other MRS metrics was observed (NAA/tCr, Cho/NAA, Cho/tCr, tNAA/tCr, and Clx/tCr (*p* > 0.05 for all, details in Table 2). The rMD_mean and rMD_25th/50th/75th values of the mutation group were significantly lower just in the adult subgroup (*p* < 0.001), but other DTI histogram parameter values were not significantly different (*p* > 0.05). In addition, rCBV_mean/25th/50th/75th were slightly higher (*p* = 0.03/0.03/0.01/0.03) and the rTTP_mean/50th were slightly lower (*p* = 0.012/0.03) in the adult mutant subgroup, and no significant differences in other PWI metrics were shown, including rCBF‐, rMTT‐, and rTTP‐related histogram parameters. For the two metrics of tumor size, no significant difference was found (*p* > 0.05). Multimodal MR images of H3 K27M mutant and wild‐type gliomas see in Figure S1. Besides, these group differences of rADC‐related histogram metrics and Ins/tCr were also found in the validation set, please see Figure S2.

**TABLE 2:** The DWI and MRS characteristics of diffuse midline gliomas

Variables	Patients’ age <18 years				Patients’ age ≥18 years			
	H3 K27M wild‐type group	H3 K27M mutant group	Statistic	*p* value	H3 K27M wild‐type group	H3 K27M mutant group	Statistic	*p* value
DWI								
rADC_M	1.92 ± 0.40	1.56 ± 0.20	2.24	0.05^†^	1.58 ± 0.19	1.50 ± 0.31	0.76	0.46^†^
rADC_15th	1.34 (1.31,1.43)	1.15 (1.07, 1.26)	70	0.005*	1.23 ± 0.14	1.07 ± 0.31	2.47	0.024* ^†^
rADC_25th	1.47 (1.40, 1.57)	1.31 (1.19, 1.37)	65	0.019*	1.32 ± 0.15	1.15 ± 0.31	2.55	0.02* ^†^
rADC_50th	1.73 (1.63, 1.81)	1.50 (1.36, 1.59)	63	0.03*	1.53 ± 0.19	1.33 ± 0.31	2.16	0.046* ^†^
rADC_75th	2.04 (1.89, 2.22)	1.68 (1.57, 1.84)	63	0.03*	1.83 (1.59, 1.92)	1.60(1.43, 1.71)	200	0.06
rADC_max	3.37 ± 0.95	2.94 ± 0.82	0.98	0.35^†^	2.49 ± 0.56	3.05 ± 1.12	−1.65	0.12^†^
MRS								
Ins/tCr	1.68 ± 0.36	1.11 ± 0.43	3.4	0.003* ^†^	1.20 (0.98, 1.51)	0.80 (0.64, 1.21)	**211.5**	0.025*
NAA/tCr	0.56 ± 0.30	0.62 ± 0.32	−0.43	0.67^†^	0.60 ± 0.23	0.60 ± 0.23	0.03	0.98^†^
Cho/NAA	1.51 (0.76, 2.22)	0.96 (0.77, 1.53)	49	0.71	0.88 (0.57, 1.62)	1.11 (0.75, 1.65)	118	0.39
Cho/tCr	0.73 (0.42, 0.99)	0.65 (0.43, 0.99)	43	0.97	0.46 (0.37, 0.71)	0.57 (0.52, 0.93)	101	0.15
tNAA/tCr	0.72 (0.52, 0.80)	0.63 (0.47, 1.11)	46	0.90	0.78 ± 0.32	0.86 ± 0.30	−0.78	0.44^†^
Glx/tCr	0.96 ± 0.88	0.89 ± 0.39	0.20	0.84^†^	0.83 (0.62, 0.98)	0.97 (0.62, 1.26)	124	0.51

The bold values means *p* < 0.05.

Abbreviations: Cho, choline; DWI, diffusion‐weighted imaging; Glx, glutamine plus glutamate; Ins, myo‐Inositol; MRS, magnetic resonance spectroscopy; NAA, N‐Acetylaspartate; rADC, relative apparent diffusion coefficient; tCr, creatine plus phosphocreatine; tNAA, N‐Acetylaspartate plus N‐Acetylaspartylglutamate.

† Student's *t*‐test; nonparametric tests were performed with others; *p < 0.05.

### 3.3 Prediction models and nomograms

**FIGURE 4:**
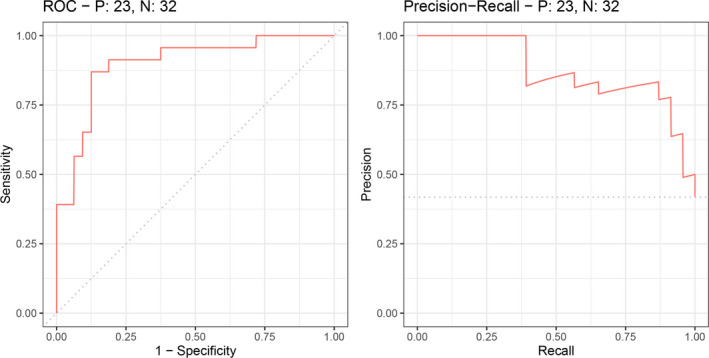
The area under the curve (AUC) was 0.898 and F1 score was 0.732 of the predictive H3 K27M status model

After nested cross‐validation of the whole cohort, five most important variables were selected from five classification models, whose average importance score was higher than 70. These variables were age, Ins/tCr, rADC_15th, rMD_50th, and rMD_75th. Then, by using the LASSO classifier, three variables were selected as potential predictors of H3 K27M mutation status, which were nonzero coefficients in the LASSO model with a lambda.1se value of 0.095. In the whole cohort, the C‐index was 0.898 (95% CI, 0.818–0.978) and the validated C‐index was 0.884 (200 resamples, Figure S3). When estimating the model performance, the AUC was 0.898 and F1 score was 0.732 for the final logistic model based on the three valuable variables for the whole cohort (see Figure 4). Moreover, the AUC was 0.976 in the validation set (see Figure S4). And the details of the nomogram on the whole cohort are shown in Figure S5.

## 4 DISCUSSION

This study not only used descriptive/qualitative information such as tumor location, but also employed quantitative MR metrics such as histogram parameters and MRS data to investigate the clinical and radiological characteristics of midline gliomas with or without H3 K27M mutation. In the subgroup analysis stratified by age, we found that Ins/tCr and rADC‐related histogram parameters were lower in the mutant group of juvenile and adult patients. We also found that the age, rMD‐, rCBV‐, and rTTP‐related histogram parameters had intergroup differences in the adult subgroup. After the rigorous process of feature selection for the whole cohort, our model revealed that age, Ins/tCr, and rADC_15th were the most valuable and effective predictors of H3 K27M mutation, with an AUC of 0.898 and a F1 score of 0.732.

In agreement with prior studies, our study also demonstrated that the H3 K27M mutation was more likely to occur in pediatric and young adult patients.^7^, ^8^, ^9^, ^22^, ^23^ Recently, a study using genetically engineered inducible mice showed that H3.3 K27M mutation in DIPGs could enhance the self‐renewal of neural stem cells (NSCs).^24^ The role of the H3.3 K27M mutation in the early stage of neoplastic initiation is congruent with clonal incidence in DIPGs, which may account for the susceptibility of pediatric patients,^24^ because neural stem cells are in different cellular states in pediatric and adult patients. It is possible that the H3.3 K27M‐dependent target genes may change with age and lead to a decline in mutation incidence when age increases. Of course, more studies on humans are needed to determine mutation pathogenesis.

Although the present study demonstrated that the H3 K27M mutation group had a relatively shorter median OS than the wild‐type group, the difference did not reach significance. There are two possible explanations for this result. The first is the small population included in survival analysis, that is, only 22 patients had complete follow‐up data. The second is the contribution from the adult patients. According to the studies of DIPG, H3 K27M mutation was associated with poor prognosis^25^ and was inoperable without cure.^26^ It is worth noting that most DIPG cases involve pediatric patients. Unlike pediatric patients, adult midline glioma patients with H3 K27M mutations did not show overall survival rates that were different from wild‐type patients.^9^, ^22^ Therefore, subgroup analysis based on a large case population would provide additional interesting information for survival analysis.

In our cohort, tumors with H3 K27M mutation were mostly located in the brainstem followed by other midline structures in juvenile and adult patients. For the H3 K27M wild‐type group, the diencephalic structure near the midline was the major location of the tumors. DIPGs with a high frequency of H3 K27M mutations (78%) are located in the brainstem, and some researchers have proposed that this midline location may have a special molecular pathway.^3^ However, these findings need a large case population to be confirmed, and the speculation needs to be verified by physiopathological and genetic studies.

Multimodal MR imaging plays an important role in the diagnosis and management of gliomas.^27^ A recent study using ADC to predict the status of H3 K27M mutation reported that several ADC values, including the ratio of minimal and peritumoral ADC values, were significantly lower in the H3 K27M mutation group than in the wild‐type group in both juvenile and adult patients.^28^ Similarly, in our study, most rADC‐related histogram values in the mutation group were also lower than those in the wild‐type group. In particular, rADC_15th was one of the most valuable parameters to predict the status of H3 K27M mutation in our study. This indicates that most H3 K27M mutant diffuse midline gliomas demonstrated low rADC compared with wild‐type. In another study, the fifth percentile obtained from cumulative ADC histograms also seemed to be a reliable histogram parameter for grading gliomas.^17^ Lower ADC values were generally considered to be associated with increased tumor cellularity^29^ and were found in high‐grade gliomas rather than in low‐grade gliomas.^30^ The mutation group showed typical features of glioblastomas (WHO grade IV),^31^ which were usually linked to lower rADC values. On this basis, we speculated that ADC‐related histogram parameters partly contribute to identifying the status of an H3 K27M mutation.

Our results also showed that the rMD_mean, rMD_25th, rMD_50th, and rMD_75th values of DTI data in the mutation group were lower than those in the wild‐type group in adult subgroup. However, these metrics had no significant differences of juvenile subgroup. DTI is a common imaging method used to visualize the displacement of white matter tracts caused by tumors, which has been applied to evaluate the tumor grade^32^ or to predict the status of the isocitrate dehydrogenase 1 (IDH1) gene.^33^ A preoperative evaluation of gliomas demonstrated that MD values of low‐grade gliomas were significantly higher than those of high‐grade gliomas.^32^ MD had a positive correlation with IDH1 status in HGG,^33^ and gliomas with IDH mutation have a better prognosis compared with IDH wild‐type gliomas^34^; and thus higher MD may be linked to a better prognosis. In our study, a similar correlation was observed in juvenile group (though no significant differences), that is, midline gliomas with an H3 K27M mutation had lower rMD and a worse prognosis than wild‐type. It was consistent with previous studies that patients with H3 K27M mutation had poor survival in juvenile subgroup but not in adult.^4^, ^22^ A study of DIPGs showed that an FA reduction may play a role in reflecting tumor extension and the values of DTI parameters are useful in the detection of early tumor infiltration.^35^ This indicates that DTI‐related histogram parameters might be effective in determining the status of an H3 K27M mutation and this deserves further investigation.

Magnetic resonance spectroscopy is another noninvasive neuroimaging method that is able to evaluate tumor metabolism. Heterogeneous metabolic patterns were found, and MRS was recommended for targeted treatment for DIPG patients in a previous longitudinal MRS study.^36^, ^37^ In the present study, low Ins/tCr was observed in the mutation group in both juveniles and adults, which mostly included highly malignant tumors based on histopathological findings. According to a study of astrocytoma, myo‐inositol levels decreased as the tumor grade increased.^38^ Therefore, low Ins/tCr may also be related to H3 K27M mutations in gliomas, which are highly malignant tumors with poor prognosis. Meanwhile, Ins/tCr was also a valuable variable of predicting H3 K27M status. Therefore, low Ins/tCr may be a potential imaging biomarker for diffuse midline gliomas with H3 K27M mutation. This should be confirmed in a large cohort.

Three variables were included in the logistic model to predict the H3 K27M mutation status, including two radiological features (rADC_15th and Ins/tCr) and one clinical variable (age of the patient). All these variables could be acquired during routine clinical examination. The model performed well in predicting the H3 K27M status with a calibrated C‐index of 0.898 (AUC was 0.898) and an F1 score of 0.732. Thus, such a model combing age, Ins/tCr, and rADC_15th could serve as a powerful and noninvasive predictive tool to evaluate a patient’s H3 K27M mutation status to optimize treatment planning in clinical conditions.

However, the results in the present study should be interpreted while considering the following limitations. The relatively small sample size, due to a small number of patients undergoing multimodal MR examinations and the exclusion of some patients, limited the statistical power of the present study. And we had no prospective cohort to verify the model performance. But we performed a retrospective validation in an independent set, which also showed good performance. Moreover, to overcome the overfitting problem of small sample size, we combined the nested cross‐validation and LASSO algorithm to efficiently reduce the dimensionality of data. A previous study recommended that the patient population of the less response group should be 10 times greater than the number of predictors in the binary response study.^39^ In our study, we had three predictors and <30 patients in the H3 K27M mutation group. Thus, future studies with a larger case population and more preoperative multimodal MR examinations are needed to validate the prediction performance. Finally, to further explore whether imaging biomarkers and prognosis are different in pediatric and adult groups, future studies should include more patients of different ages.

## 5 CONCLUSIONS

In conclusion, our study found that some quantitative MR metrics as well as clinical variables were significantly different between the H3 K27M mutation and wild‐type midline gliomas, among which age, In/tCr, and rADC showed high diagnostic power in predicting H3 K27M mutation status. However, the value of the prediction model needs to be further verified in future studies based on a larger case population.

## 6 ETHICAL APPROVAL STATEMENT

This study was approved by the Institutional Review Board (IRB) of our hospital, and the requirement for written informed consent was waived.

## CONFLICT OF INTEREST

None.

## AUTHOR CONTRIBUTIONS

*Conceptualization*: Xr Su, Yh Liu, Q Yue, and Qy Gong; *Data curation*: Xr Su, Yh Liu, Q Yue, and Qy Gong; *Formal analysis*: Xr Su, Yh Liu, Hq Sun, Wn Wang, N Chen, Q Yue, and Qy Gong; *Funding acquisition*: Yh Liu, Q Yue, and Qy Gong; *Investigation*: Xr Su and Yh Liu; *Methodology*: Hq Sun and Wn Wang; *Project administration*: Yh Liu, Q Yue, and Qy Gong; *Resources*: Q Yue and Yanhui Liu; *Software*: Hq Sun and Hy Wang; *Supervision*: Yh Liu, Q Yue, and Qy Gong; *Validation*: Sm Zhang, Xb Yang, Xy Wan, and Qy Tan; *Visualization*: Wn Wang and Sm Zhang; *Writing*‐*original draft*: Xr Su and Yh Liu; *Writing*‐*review & editing*: Q Yue and Qy Gong.

## Supporting information

Supplementary Material

## Data Availability

The data that support the findings of this study are available upon request from the corresponding author. The data are not publicly available due to privacy or ethical restrictions.
