# Supplementary material for: Multimodal MR imaging signatures to identify brain diffuse midline gliomas with H3 K27M mutation
Source: Cancer Med. 2021 Dec 24;11(4):1048–58. doi: 10.1002/cam4.4500 (PMC8855915; doi:10.1002/cam4.4500)
Supplement: Supplementary file 1 — Supplementary Material [file CAM4-11-1048-s001.zip › Supporting Information/Supplementary Table.docx]

**Supplemental Table S1 The Radiographical Characteristics of Diffuse Midline Gliomas**

| Variables | Patients’ Age < 18 years | | | | Patients’ Age ≥ 18 years | | | |
| --- | --- | --- | --- | --- | --- | --- | --- | --- |
|  | H3 K27M wild-type group | H3 K27M mutant group | Statistic | P value | H3 K27M wild-type group | H3 K27M mutant group | Statistic | P value |
| **DTI** |  |  |  |  |  |  |  |  |
| rFA_M | 0.54 ± 0.08 | 0.50 ± 0.11 | 0.85 | 0.41† | 0.55 ± 0.09 | 0.50 ± 0.14 | 1.26 | 0.23† |
| rFA 25th | 0.31 ± 0.12 | 0.31 ± 0.11 | -0.02 | 0.99† | 0.35 ± 0.07 | 0.31 ± 0.12 | 1.22 | 0.24† |
| rFA_50th | 0.47 ± 0.11 | 0.44 ± 0.13 | 0.48 | 0.63† | 0.51 ± 0.09 | 0.45 ± 0.15 | 1.21 | 0.24† |
| rFA_75th | 0.71 ± 0.09 | 0.63 ± 0.14 | 1.37 | 0.19† | 0.70 ± 0.12 | 0.64 ± 0.18 | 1.07 | 0.30† |
| rFA_max | 1.38 ± 0.23 | 1.31 ± 0.29 | 0.56 | 0.58† | 1.22 (1.07, 1.53) | 1.13 (0.94, 1.13) | 166 | 0.34 |
| rMD_M | 1.24 ± 0.21 | 1.28 ± 0.27 | -0.34 | 0.73† | 1.42 (1.35, 1.58) | 1.28 (1.25, 1.38) | 206 | **0.019*** |
| rMD_25th | 1.05 ± 0.22 | 1.08 ± 0.24 | -0.30 | 0.77† | 1.22 ± 0.13 | 1.09 ± 0.17 | 2.26 | **0.03*†** |
| rMD_50th | 1.20 ± 0.22 | 1.24 ± 0.25 | -0.41 | 0.69† | 1.36 (1.31, 1.49) | 1.21 (1.13, 1.37) | 215 | **0.008*** |
| rMD_75th | 1.37 ± 0.24 | 1.42 ± 0.28 | -0.43 | 0.67† | 1.59 (1.50, 1.75) | 1.37 (1.30, 1.53) | 225 | **0.003*** |
| rMD_max | 1.95 (1.80, 2.23) | 1.91 (1.78, 3.36) | 37 | 0.93 | 2.36 (2.01, 2.64) | 2.86 (1.93, 3.73) | 110 | 0.34 |
| **PWI** |  |  |  |  |  |  |  |  |
| rCBV_M | 1.65 (1.32, 2.18) | 1.63 (1.34, 2.83) | 42 | 0.90 | 1.65 (1.39, 2.31) | 2.12 (2.02, 2.42) | 79 | **0.03*** |
| rCBV_25th | 1.03 ± 0.59 | 1.05 ± 0.69 | -0.08 | 0.94† | 0.79 (0.63, 1.11) | 1.10 (1.02, 1.27) | 79 | **0.03*** |
| rCBV_50th | 1.30 (1.01, 1.82) | 1.21 (0.85, 2.40) | 48 | 0.77 | 1.31 (0.98, 1.86) | 1.69 (1.56, 2.10) | 71 | **0.01*** |
| rCBV_75th | 1.95 (1.66, 2.84) | 1.97 (1.57, 3.56) | 43 | 0.97 | 2.07 (1.69, 2.86) | 2.73 (2.52, 3.26) | 77 | **0.03*** |
| rCBV_85th | 2.73 (1.99, 3.47) | 2.55 (2.31, 4.39) | 40 | 0.77 | 2.67 (2.20, 3.62) | 3.57 (3.10, 3.96) | 89 | 0.07 |
| rCBV_max | 5.92 (4.21, 7.61) | 7.48 (5.78, 9.32) | 31 | 0.30 | 5.91 ± 2.40 | 6.74 ± 1.32 | -1.35 | 0.19† |
| rCBF_M | 1.94 ± 1.30 | 1.43 ± 0.47 | 1.06 | 0.32† | 1.54 (1.09, 2.22) | 1.81 (1.23, 2.09) | 138 | 0.85 |
| rCBF_25th | 0.86 ± 0.46 | 0.68 ± 0.297 | 0.97 | 0.35† | 0.77 (0.57, 0.96) | 0.83 (0.72, 1.02) | 129 | 0.63 |
| rCBF_50th | 1.44 (0.75, 1.72) | 1.14 (0.80, 1.16) | 56 | 0.34 | 1.27 (0.95, 1.63) | 1.40 (1.07, 1.52) | 139 | 0.88 |
| rCBF_75th | 2.62 ± 1.98 | 1.67 ± 0.56 | 1.32 | 0.22† | 1.93 (1.33, 2.83) | 2.16 (1.56, 2.47) | 141 | 0.93 |
| rCBF_max | 6.91 ± 4.89 | 5.69 ± 2.43 | 0.65 | 0.53† | 5.36 ± 2.76 | 5.51 ± 1.84 | -0.18 | 0.85† |
| rMTT_M | 1.27 ± 0.28 | 1.27 ± 0.19 | -0.04 | 0.97† | 1.13 (1.03, 1.29) | 1.17 (1.05, 1.31) | 134 | 0.75 |
| rMTT_25th | 0.95 ± 0.15 | 0.93 ± 0.24 | 0.27 | 0.79† | 0.96 (0.88, 1.00) | 0.93 (0.86, 1.05) | 146 | 0.96 |
| rMTT_50th | 1.17 ± 0.26 | 1.19 ± 0.21 | -0.16 | 0.87† | 1.08 (0.98, 1.21) | 1.09 (0.98, 1.28) | 139 | 0.88 |
| rMTT_75th | 1.49 (1.21, 1.85) | 1.54 (1.38, 1.78) | 38 | 0.65 | 1.20 (1.12, 1.52) | 1.25 (1.16, 1.63) | 125 | 0.53 |
| rMTT_max | 2.17 (1.88, 2.71) | 2.26 (2.04, 2.69) | 38 | 0.65 | 2.11 (1.82, 2.54) | 2.09 (1.84, 2.42) | 147 | 0.93 |
| rTTP_M | 1.05 (0.98, 1.19) | 1.04 (1.03, 1.06) | 43 | 0.97 | 1.05 ± 0.06 | 1.00 ± 0.04 | 2.66 | **0.012**^*^† |
| rTTP_25th | 1.02 (0.91, 1.05) | 1.00 (0.99, 1.01) | 52 | 0.54 | 0.99 (0.95, 1.01) | 0.94 (0.92, 1.00) | 186.5 | 0.16 |
| rTTP_50th | 1.07 (1.00, 1.16) | 1.01 (1.00, 1.09) | 52 | 0.54 | 1.05 ± 0.07 | 1.01 ± .04 | 2.16 | **0.03***† |
| rTTP_75th | 1.11 (1.00, 1.27) | 1.08 (1.06, 1.10) | 45 | 0.97 | 1.10 ± 0.09 | 1.05 ± 0.05 | 1.87 | 0.07† |
| rTTP_max | 1.37 (1.14, 1.67) | 1.28 (1.26, 1.84) | 46 | 0.90 | 1.23 (1.17, 1.43) | 1.33 (1.26, 1.41) | 114 | 0.32 |
| Tumor size |  |  |  |  |  |  |  |  |
| Longest 1 | 3.69 (2.55, 4.36) | 4.45 (3.68, 4.83) | 34 | 0.43 | 2.90 ± 0.94 | 2.33 ± 0.69 | 2.05 | 0.05† |
| Longest 2 | 3.03 (2.42, 3.42) | 3.35 (3.15, 3.78) | 26 | 0.15 | 2.71 (2.33, 3.76) | 2.27 (1.91, 3.65) | 194.5 | 0.09 |

Notes: †, Student’s T-test; nonparametric tests were performed with others; *, P<0.05

PWI, Perfusion weighted imaging; rCBV, relative cerebral blood volume; rCBF, relative cerebral blood flow; rMTT, relative mean transit time; rTTP, relative time to peak; longest 1, the longest transverse diameter of hyperintense area on FLAIR image; longest 2, the longest anteroposterior diameter of hyperintense area on FLAIR image.
