# Supplementary figures and images for: Multimodal MR imaging signatures to identify brain diffuse midline gliomas with H3 K27M mutation
Source: Cancer Med. 2021 Dec 24;11(4):1048–58. doi: 10.1002/cam4.4500 (PMC8855915; doi:10.1002/cam4.4500)

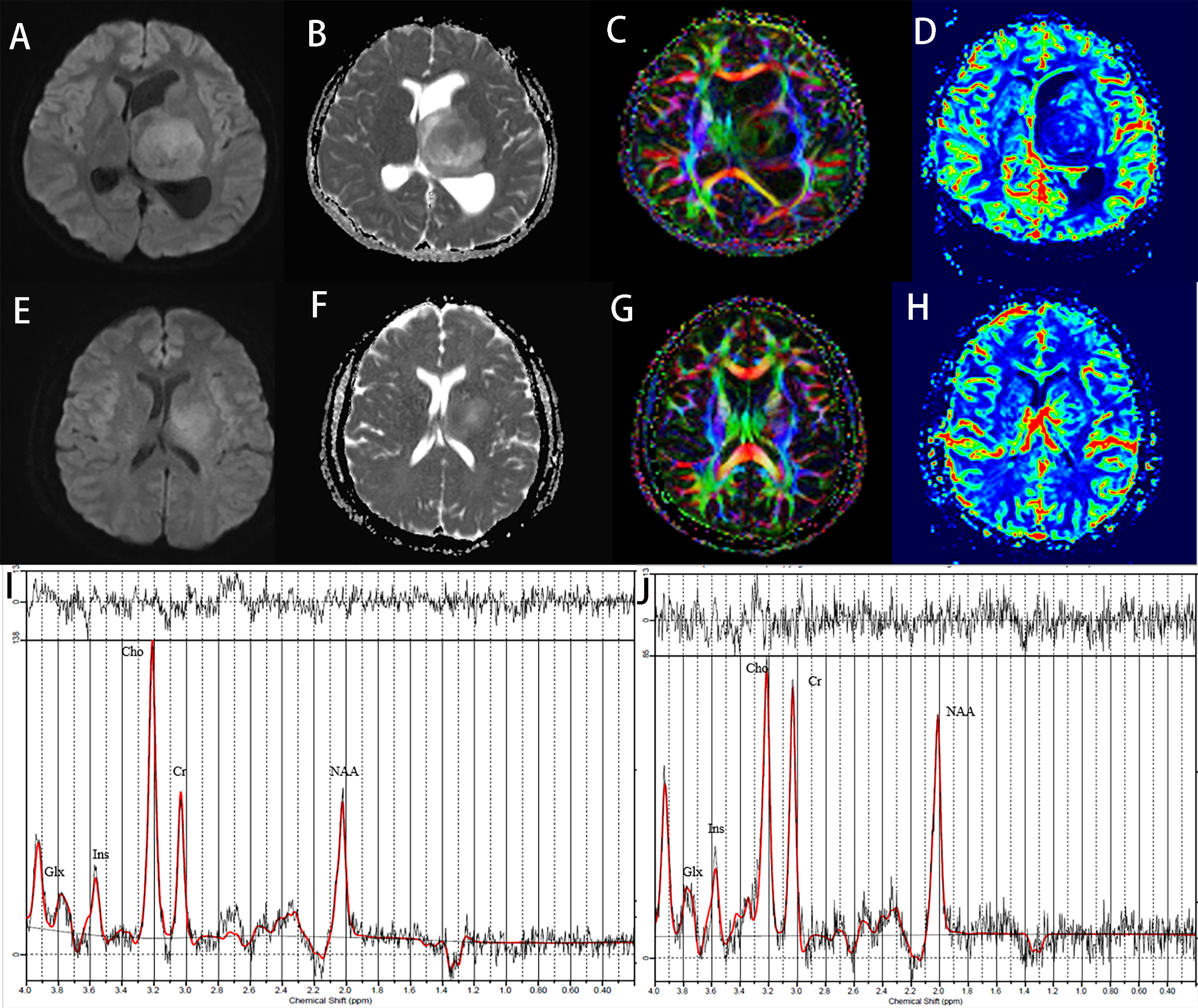

Supplement: Supplementary file 1 — Supplementary Material [file CAM4-11-1048-s001.zip › Supporting Information/Supplementary Figure S1.tif]

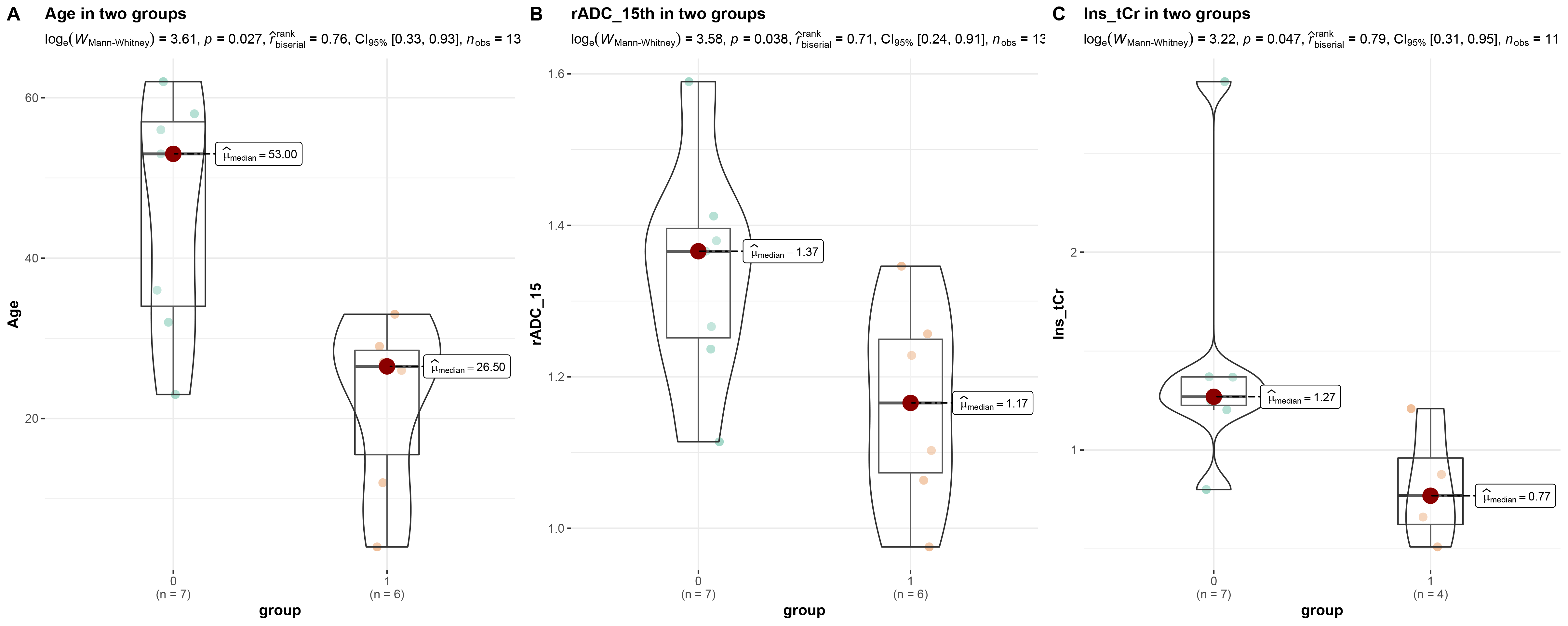

Supplement: Supplementary file 1 — Supplementary Material [file CAM4-11-1048-s001.zip › Supporting Information/Supplementary Figure S2.tif]

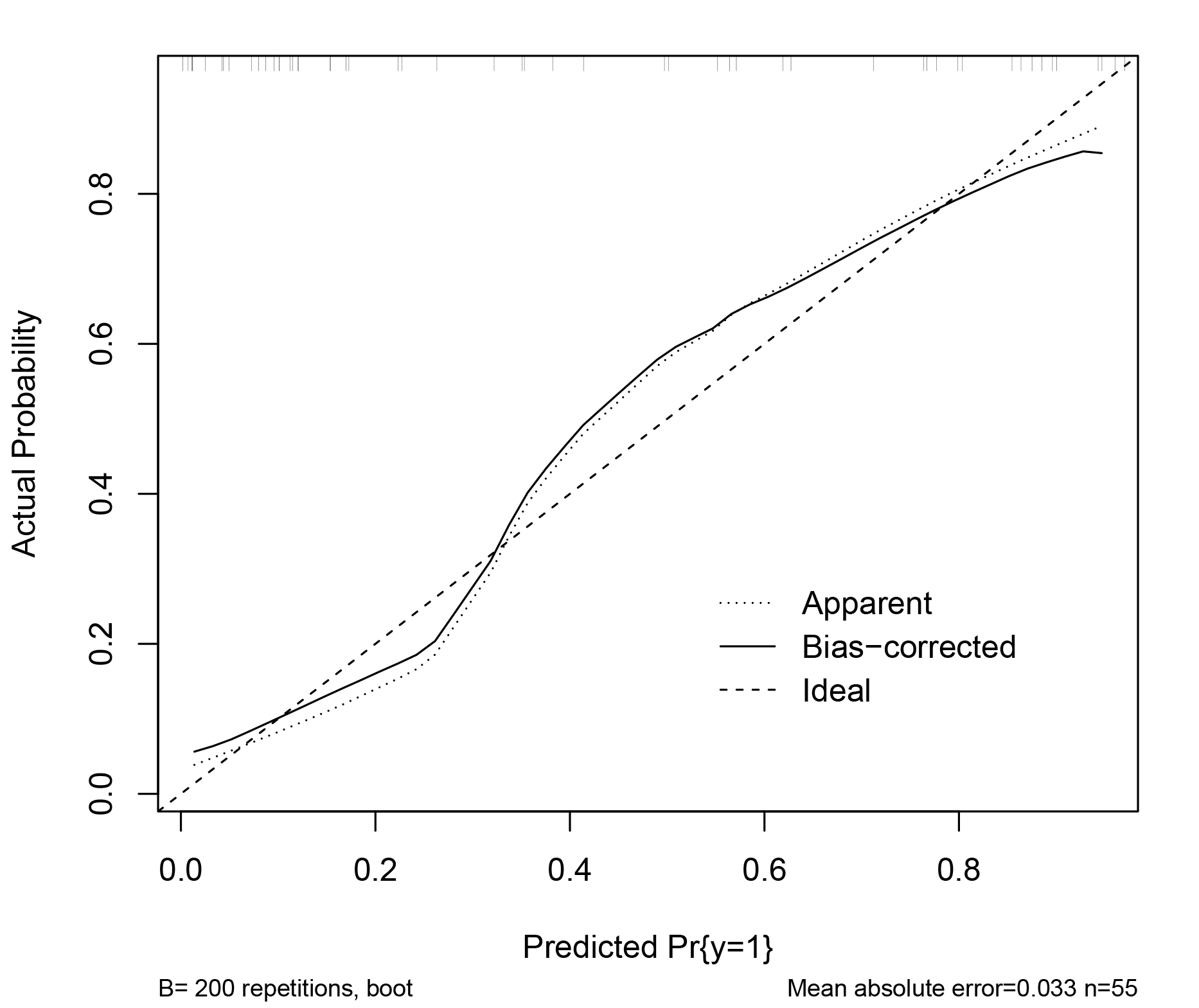

Supplement: Supplementary file 1 — Supplementary Material [file CAM4-11-1048-s001.zip › Supporting Information/Supplementary Figure S3.tif]

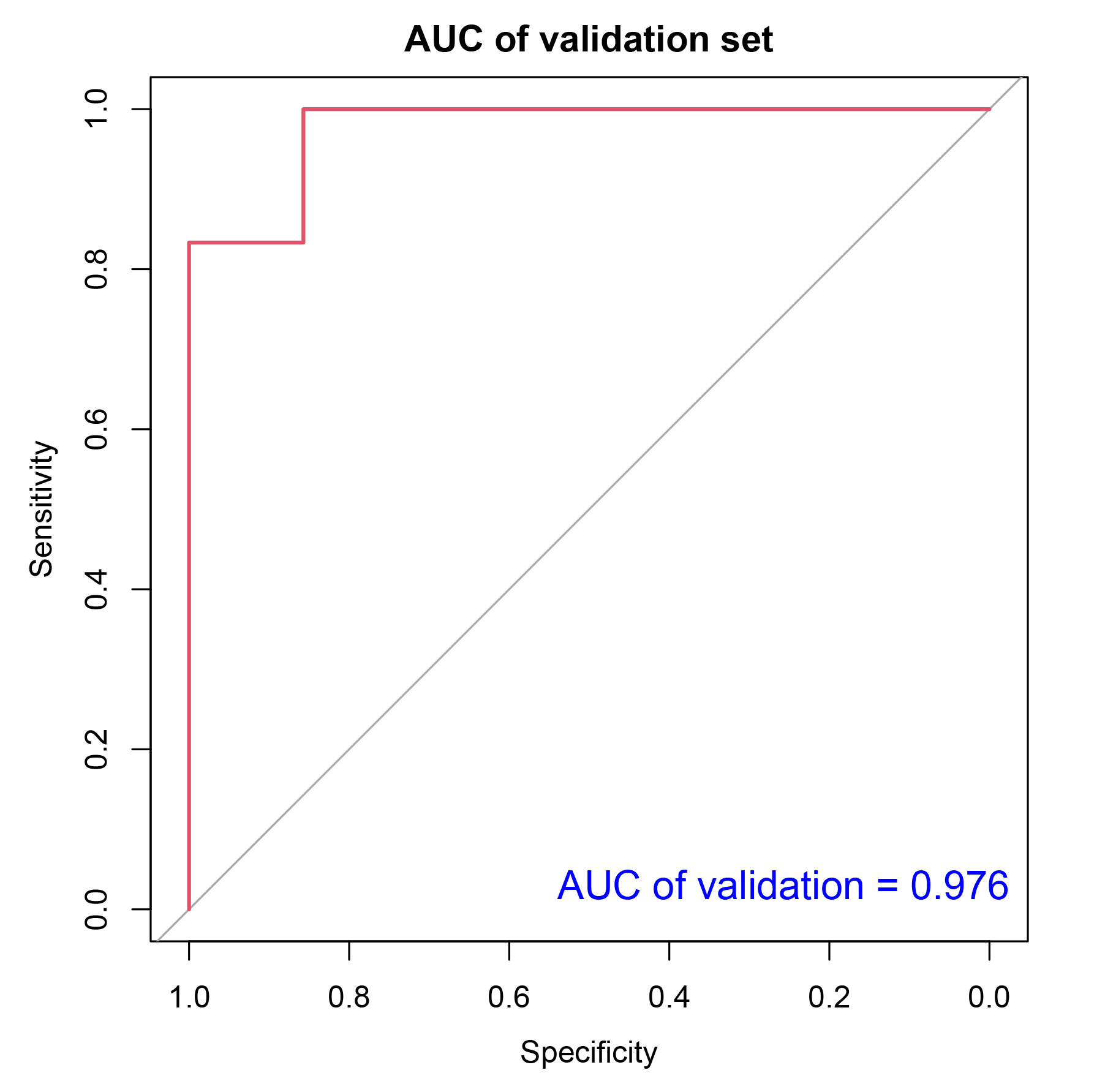

Supplement: Supplementary file 1 — Supplementary Material [file CAM4-11-1048-s001.zip › Supporting Information/Supplementary Figure S4.tif]

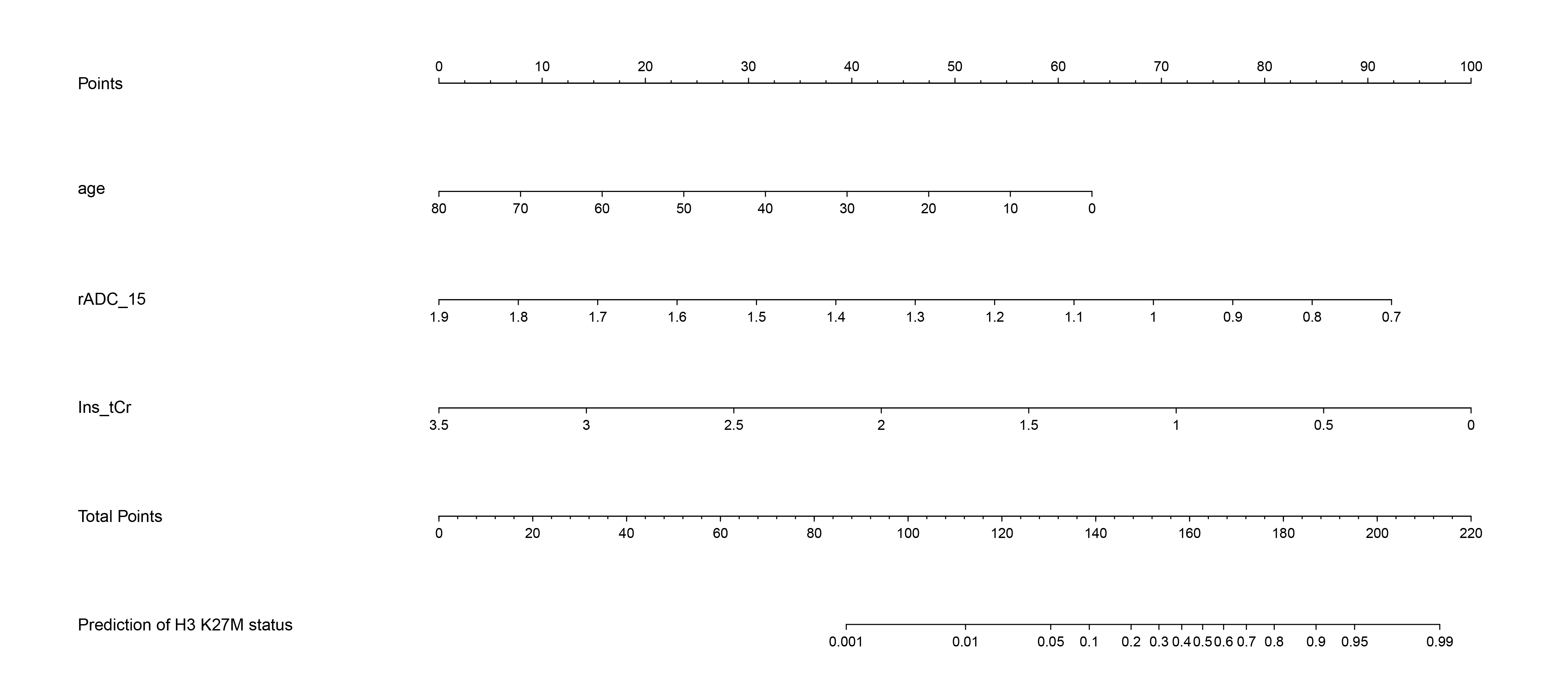

Supplement: Supplementary file 1 — Supplementary Material [file CAM4-11-1048-s001.zip › Supporting Information/Supplementary Figure S5.tif]
